# Differential Spatial Expression and Subcellular Localization of CtBP Family Members in Rodent Brain

**DOI:** 10.1371/journal.pone.0039710

**Published:** 2012-06-22

**Authors:** Diana Hübler, Marija Rankovic, Karin Richter, Vesna Lazarevic, Wilko D. Altrock, Klaus-Dieter Fischer, Eckart D. Gundelfinger, Anna Fejtova

**Affiliations:** 1 Department of Neurochemistry and Molecular Biology, Leibniz Institute for Neurobiology, Magdeburg, Germany; 2 Institute of Biochemistry and Cell Biology, Otto-von-Guericke University, Magdeburg, Germany; 3 German Center for Neurodegenerative Disorders (DZNE), Magdeburg Branch, Magdeburg, Germany; University of Salamanca- Institute for Neuroscience of Castille and Leon and Medical School, Spain

## Abstract

C-terminal binding proteins (CtBPs) are well-characterized nuclear transcriptional co-regulators. In addition, cytoplasmic functions were discovered for these ubiquitously expressed proteins. These include the involvement of the isoform CtBP1-S/BARS50 in cellular membrane-trafficking processes and a role of the isoform RIBEYE as molecular scaffolds in ribbons, the presynaptic specializations of sensory synapses. CtBPs were suggested to regulate neuronal differentiation and they were implied in the control of gene expression during epileptogenesis. However, the expression patterns of CtBP family members in specific brain areas and their subcellular localizations in neurons in situ are largely unknown. Here, we performed comprehensive assessment of the expression of CtBP1 and CtBP2 in mouse brain at the microscopic and the ultra-structural levels using specific antibodies. We quantified and compared expression levels of both CtBPs in biochemically isolated brain fractions containing cellular nuclei or synaptic compartment. Our study demonstrates differential regional and subcellular expression patterns for the two CtBP family members in brain and reveals a previously unknown synaptic localization for CtBP2 in particular brain regions. Finally, we propose a mechanism of differential synapto-nuclear targeting of its splice variants CtBP2-S and CtBP2-L in neurons.

## Introduction

**Figure 1:**
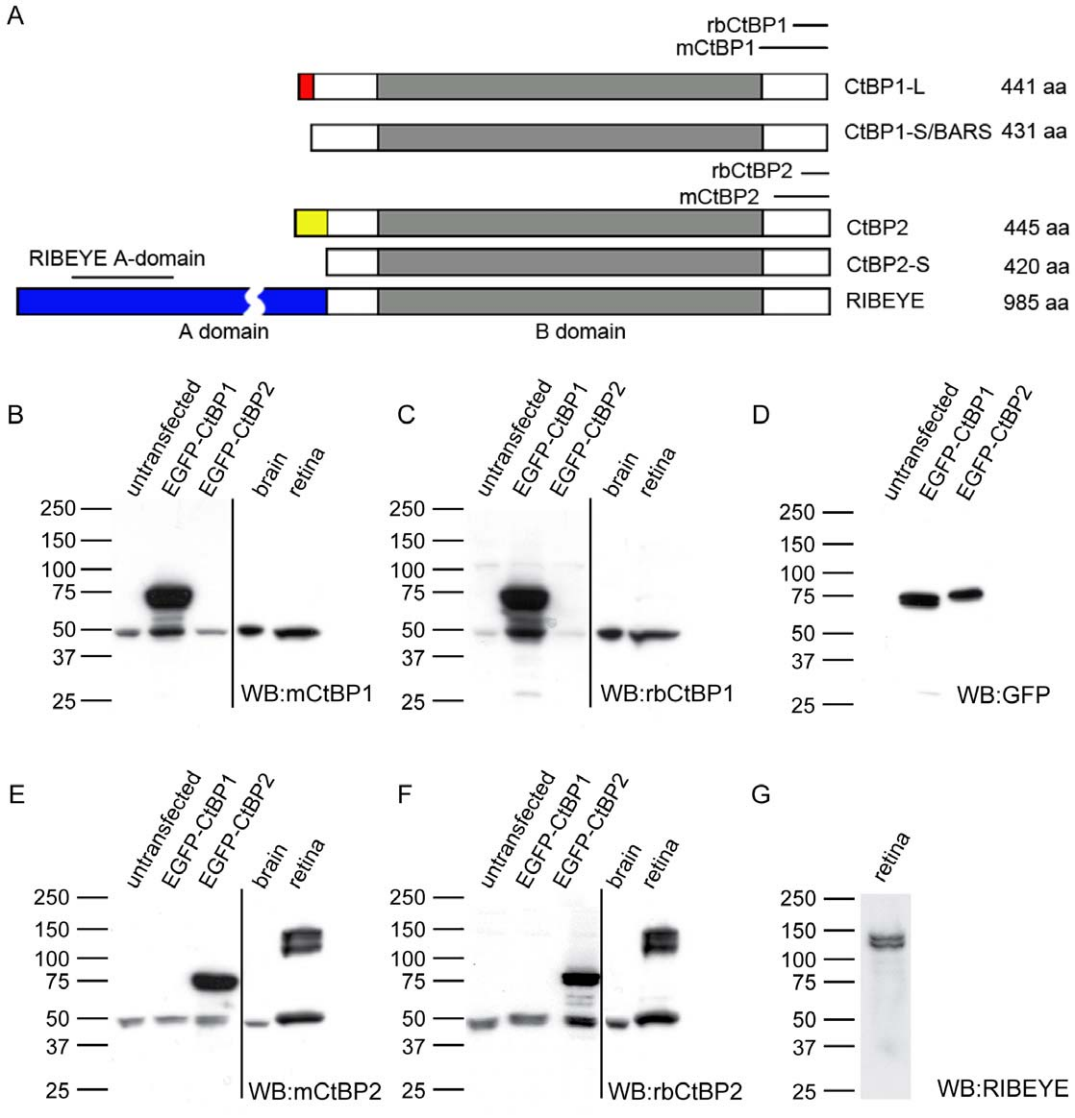
Specificity test of antibodies against CtBP1 and CtBP2. Schematic representation of domain structure of members of CtBP protein family is shown in A. The region in grey represents the high homology region shared by all family members. The red, yellow and blue marked regions depict unique N-terminal sequence expressed in CtBP1-L, CtBP2 and RIBEYE respectively. The positions of antigens used for generating antibodies used in this study are depicted as bars above the corresponding sequence. To test specificity of available antibodies, the indicated samples were tested by Western blot analysis using mouse monoclonal or rabbit polyclonal antibodies against CtBP1 or CtBP2 (B, C, E, F), rabbit polyclonal antibody against GFP (D) and RIBEYE specific antibody from rat (G). Bars and numbers indicate position and size (in kDa) of the molecular weight markers.

C-terminal binding proteins (CtBPs) were originally described and extensively studied as transcriptional co-repressors, indispensable for animal development and acting by repressing activity of large number of transcriptional factors [1]. In the past years also cytoplasmic functions for CtBP protein family members were suggested, such as dynamin-independent membrane fission during intracellular trafficking [2], fission of COPI vesicles [3], Golgi partitioning during mitosis [4] or scaffolding of ribbon synapses [5]. Alternative transcription initiation and splicing of the two genes for CtBPs results in expression of several CtBP isoforms that have some specific but also overlapping functions (Fig. 1A). The CtBP1 gene locus codes for two protein products CtBP1-S (where S stands for short; also named BARS50) and CtBP1-L (L stands for long). They are translated from mRNAs with distinct ATG-coding first exons generated by an alternative splicing and differ thus in their N-termini [6], [7]. Both CtBP1 isoforms display largely overlapping sub-cellular localization [8] and share most probably similar functions in regulation of gene expression and membrane trafficking processes [9]. The CtBP2 gene locus codes for three isoforms. The two isoforms CtBP2-S [8] and CtBP2-L [10] derived by alternative splicing from the same transcript are highly homologous to the isoforms of CtBP1 proteins. To date CtBP2-S and CtBP2-L were only described to function as nuclear transcriptional regulators. The third isoform, called RIBEYE, is expressed from an alternative promoter, active only in ribbon synapse containing neurons such as retinal photoreceptors and bipolar cells, hair cells of cochlea or pinealocytes of epiphysis [5], [11], [12]. RIBEYE has a large unique N-terminal A-domain, which is unrelated to other CtBP isoforms and a B-domain that is identical with CtBP2. It is a major structural component of synaptic ribbons, which are characterized by a high rate of tonic neurotransmitter release mediated by continuous synaptic vesicle exocytosis [5], [13].

CtBPs interact with a wide array of transcription factors; their deletion in Drosophila is not compatible with proper embryonic development [14]. CtBP1 knockout mice are viable and fertile, even though they are smaller and show higher juvenile mortality. The CtBP2 deletion is, however, lethal and leads to severe defects in early embryonic development [15]. In situ hybridization studies demonstrated ubiquitous embryonic expression of both CtBP1 and CtBP2, with notably strong expression of both proteins in the nervous system [16]. Accordingly, severe developmental defects of nervous system were found in double mouse mutants for CtBP1 and CtBP2. Interestingly, CtBP1 and CtBP2 are expressed also in terminally differentiated neurons of the adult mouse brain (Allen Mouse Brain Atlas [Internet]. Seattle (WA): Allen Institute for Brain Science. ©2009. Available from: http://mouse.brain-map.org), suggesting a role of CtBPs beyond the regulation of cell differentiation. Indeed, transcriptional repression by CtBPs regulates gene expression in epileptogenesis [17], suggesting a possible involvement of these proteins also in activity-dependent gene expression, which is indispensable for higher brain function including learning and memory. Moreover, we have shown previously a presynaptic localization of CtBP1 in cultured hippocampal neurons [18], what suggests a function of this protein apart of transcriptional regulation. Thus, investigations of CtBP functions in the brain are of high interest. As a prerequisite for these studies it has to be known how the members of CtBP family are expressed in different brain areas and what is their subcellular localization in brain neurons. To this end, we have undertaken careful analysis of expression patterns for both CtBP1 and CtBP2 in the mouse brain at the microscopic and at the ultra-structural level and analysed their expression in neuronal nuclei and synapses using immunohistochemical and biochemical approaches. Our study reveals differential regional and subcellular expression patterns for the two CtBP family members. Moreover, we describe here a previously unknown synaptic localization for CtBP2 and propose a mechanism of differential sub-cellular targeting of its splice variants CtBP2-S and CtBP2-L in neurons.

## Materials and Methods

### Animals

Brains of adult C57/BL6 mice of mixed sex were used in all biochemical experiments and for preparation of brain slices. Neuronal cultures were prepared from hippocampi of embryonic day 18 (E18) Wistar rats (strain: RjHan: WI; Elevage Janvier, France). All experiments were carried out in accordance with the European Committees Council Directive (86/609/EEC) and approved by the local animal care committee (Landesverwaltungsamt Sachen-Anhalt, AZ: 42502/2-988 IfN).

### Antibodies

The list of primary antibodies used in the study is provided in Table 1. The position of epitopes of antibodies against CtBPs used in the study are depicted in Fig. 1A. The specificity and cross-reactivity of used mouse and rabbit antibodies against CtBP1 and CtBP2 was tested and is shown in Fig. 1.

**Table 1:** List of antibodies.

antibody	immunogen	manufacturer/ citation	catalog number	species	monoclonal or polyclonal	dilution
anti-Bassoon	recombinant rat Bassoon	Stressgen	VAM-PS003	mouse	mc	IF, WB: 1∶1000
anti-Bassoon	recombinant rat Bassoon	[34]		rabbit	pc	IF: 1∶1000
anti-CtBP1	mouse CtBP1 residues 345–441	BD Trans Lab	612042	mouse	mc	IF, EM: 1∶1000; WB: 1∶5000
anti-CtBP2	mouse CtBP residues 361–445	BD Trans Lab	612044	mouse	mc	WB: 1∶2000
anti-CtBP2	rat CtBP2 residues 431–445	SYSY	193 003	rabbit	pc	IF, EM, WB: 1∶2000
anti-GAD65	C-terminal epitope	Abcam	ab26113	rabbit	mc	IF: 1∶1000
anti-GAPDH	human GAPDH residues 1–16	Abcam	ab37168	rabbit	pc	WB: 1∶3000
anti-GFP clone B34	recombinant GFP protein	Covance/Babco	MMS-118P	mouse	mc	WB: 1∶20000
anti-NeuN	nuclear NeuN mouse protein	Milipore	MAB377	mouse	mc	WB: 1∶100
anti-Piccolo	recombinant rat Piccolo	[35]		guinea pig	pc	WB: 1∶2000
anti –PSD95 clone K28/43	human PSD95 residues 77–299	Upstate	05–494	mouse	mc	WB: 1∶1000
anti-RIBEYEA domain	rat RIBEYE residues 101–207	SYSY	192 103	rabbit	pc	WB: 1∶250
anti- Synaptophysin	human synaptophysin residues 301–313	SYSY	101 002	rabbit	pc	WB: 1∶1000

### Subcellular fractionation of mouse brain

Synaptosomes and nuclei from whole brain were obtained from three mice brain, each. In total three independent fractionations were performed for samples from whole brain.

Due to low initial tissue amount the cortices and cerebella of three mice brains were pooled for preparation of synaptosomes and nuclei from cortex and cerebellum.

Preparation of synaptosomes was performed as previously described [19] with minor modifications. Briefly, one mouse brain (∼500 mg total wet weight) was homogenized in 10 ml of homogenization buffer (0.32 M sucrose, 10 mM TrisHCl pH 7.4, Complete mini protease inhibitor (Roche)). Homogenate was then spun at 800 g for 10 min and supernatant (S1) was brought to a final sucrose concentration of 1.5 M by the addition of 2 M sucrose solution. To obtain the synaptosomes, the supernatant S1 was overlaid with 1.25 M and 1 M sucrose cushions and spun at 100000 g for 2 h.

The preparation of nuclei was performed with the CelLytic™ NuCLEAR™ Extraction Kit (Sigma) according to the manufacturer's protocol. Briefly, one mouse brain (∼500 mg total wet weight) was homogenized in 1× isotonic lysis buffer (10 mM Tris HCl pH 7.5, 2 mM MgCl_2_, 3 mM CaCl_2_ and 0.3 M sucrose, 0.1 M DTT and protease inhibitor cocktail). The homogenate was then spun at 10000 g for 20 min and crude nuclear pellet was resolved in Extraction buffer (20 mM HEPES pH 7.9, 1.5 mM MgCl_2_, 0.42 M NaCl, 0.2 mM EDTA and 25% (v/v) Glycerol, 0.1 M DTT and protease inhibitor cocktail). To obtain the nuclear protein extract, the pellet was agitated at medium speed for 30 min at 4°C and afterwards centrifuged for 5 min at 21000 g. Nuclear proteins were afterwards found in the supernatant. Homogenate, synaptosomes and nuclei fraction were concentrated using trichloroacetic acid (TCA) precipitation.

The preparation of post-nuclear crude membrane fraction (P2) of brain regions was performed as follows: one mouse brain was separated into eight different brain regions. These are cortex, cerebellum, olfactory bulb, hippocampus, striatum, diencephalon, midbrain and pons and medulla oblongata. In total, four mice brains were used. Cortex and cerebellum (total wet weight: cortex, 0.17–0.19 g; cerebellum, ∼0.06 g) were homogenized in 2 ml of homogenization buffer (see synaptosomal preparation), and all other regions (total wet weight: striatum, 0.01 g; hippocampus, 0.01 g; diencephalon, 0.04–0.05 g; midbrain, 0.03–0.04 g; olfactory bulb, 0.01–0.02 g; pons and medulla oblongata, 0.03–0.05 g) were homogenized in 1 ml of homogenization buffer to obtain homogenate fractions (H). The homogenates were then spun at 800 g for 10 min, and the resulting supernatant S1 was spun again at 11000 g for 15 min to obtain P2 fraction of each brain region examined. The P2 fractions were resolved in 1× SDS sample buffer (2× SDS sample buffer: 500 mM Tris HCl, pH 8.5, 20% (v/v) glycerol, 4% (w/v) SDS, 1 mM EDTA; 0.001% bromphenol blue, 5% β-mercaptoethanol), incubated at 95°C for 5 min, centrifuged for 5 min at 16000 g and the supernatants were then transferred into new reaction tubes. Equal amounts of proteins from homogenate, synaptosomes, P2 and nuclei fraction (10 µg/fraction) were determined using the colorimetric amido-black assay.

For retina homogenate two retinae were isolated from an adult Wistar rat and homogenized in 200 µL of homogenization buffer (0.32 M sucrose, 4 mM Hepes, pH 7.5) with freshly added protease inhibitors Complete mini (Roche). The sample was diluted to 1 ml with homogenization buffer and proteins were precipitated using TCA. Protein pellet was air-dried, resolved in 150 µL of 2× SDS sample buffer and left overnight at 4°C with vigorous shaking. Obtained retina homogenate was then incubated for 10 min at 95°C and 2.5 µL were loaded per line on SDS gels and tested by Western blot analysis with specific antibodies.

### Cell culture and transfections

Primary cultures of rat hippocampal neurons were prepared as described previously [20]. Briefly, cells from embryonic day 18 rat brains dissociated with trypsin were plated onto poly-D-lysine-coated glass coverslips at low density (15000 cells/cover slip, diameter 18 mm) in Dulbecco's modified eagle medium (DMEM) containing 10% fetal calf serum (FCS), antibiotics (100 U/ml penicillin, 100 µg/ml streptomycin) and 0.8 mM glutamine. After 1–2 h at 37°C, the coverslips were transferred onto the 70–80% confluent monolayer of astrocytes and the medium was exchanged with Neurobasal medium (Gibco) including B27, antibiotics and glutamine. Neurons were transfected using the calcium phosphate method as described [21] on day in vitro 3 and analyzed 11 days later.

Human embryonic kidney (HEK293T, ATTC, Manassas, VA) cells were grown in DMEM supplemented with 10% FCS and transfected with 25 µg pEGFP-CtBP1 or pEGFP-CtBP2 using the calcium phosphate method. For this the DNA was resolved in transfection solution A (500 mM CaCl_2_ in ultrapure water, sterile filtered and stored at 4°C), transfection solution B was added (140 mM NaCl, 50 mM HEPES, 1.5 mM Na_2_PO_4_ in ultrapure water, sterile filtered and stored at 4°C) and after 1 min the formed precipitates were added to the cells. The cells were incubated for 4 h before media was exchanged against fresh DMEM media. Cells were lysed with 1 ml of ice-cold lysis buffer (50 mM Tris HCl pH 7.4, 0.5% Triton X-100, 10% (v/v) glycerol, 100 mM NaCl, 1.5 mM MgCl_2_) with freshly added protease inhibitors 24 h after transfection. Lysate was centrifuged for 10 min at maximum speed and cleared supernatant was subjected to Western blot analysis.

All cells were maintained at 37°C in a humidified incubator with 5% CO_2_.

### Pre-embedding immuno-electron microscopy

Male adult mice were deeply anaesthetized using a mixture of Ketavet (Parke-Davis) and Domitor (Pfizer). Animals were perfused transcardially with 0.9% NaCl for 1 min followed by 4% formaldehyde (FA) in 0.1 M phosphate buffer (PB, pH 7.4) for 12 min. The brains were removed from the skulls and post-fixed in 4% FA in PB over night at 6°C. After fixation, brains were rinsed in 0.1 M PB, sagittal sections were cut on a vibratome (60 µm) and collected in phosphate-buffered saline (PBS). Sections were cryo-protected by incubation in a solution of 1 M sucrose in PB (60 min) and freeze-thawed for 3 times. The free-floating sections were washed in PBS, and then treated with 50% methanol and 1% H_2_O_2_ in PBS for 20 min. After washing in PBS, the sections were incubated in a solution containing 10% normal goat serum (NGS) for 60 min followed by the primary antibody (mouse anti-CtBP1 or rabbit anti-CtPB2,) in the same solution supplemented with 0.1% sodium azide for 72 h at 6°C. After washing in PBS and incubation in PBS containing 0.2% bovine serum albumin (PBS-A, 1 h), the sections were incubated with biotinylated secondary goat-anti-mouse or goat-anti-rabbit antibody (Vector, 1∶2000 in PBS-A) for 20 h at room temperature. The sections were washed again, pre-incubated in PBS-A and further incubated for 4 h with an ABC-complex (Vector ABC kit, 1∶1000) in PBS-A. After washing in PBS and 0.05 M Tris HCl buffer (pH 7.6) activity of bound peroxidase was visualized by incubation in a solution containing 1.4 mM DAB and 0.013% H_2_O_2_ in 0.05 M Tris/HCl buffer (4 min). To stop the reaction the sections were washed in PBS, then transferred in 0.1 M cacodylate buffer and stored overnight at 6°C. After washing in cacodylate buffer (2 times) the sections were fixed for 60 min in 1% OsO_4_ in 0.1 M cacodylate buffer, dehydrated in a graded ethanol series including a 45 min block staining with 2% uranyl acetate in 70% ethanol, incubated in propylene oxide (2×10 min), transferred in Durcupan, incubated overnight at room temperature, flat embedded in Durcupan and polymerized. Ultrathin sections (70 nm) of the cerebellar cortex were made with an Ultracut UC6 (Leica) and examined on a Zeiss EM 900. Pictures were taken with a 2k-CDD-camera (TRS).

### Western blot analysis

Samples from homogenate, synaptosomes, P2 and nuclei fractions (10 µg protein/sample) or whole-cell lysates from transfected HEK293 cells, rat brain and retina homogenate (for antibody testing) were separated using one-dimensional Tris-glycine 5–20% gradient SDS-PAGE and then electro-transferred to PVDF membrane (Millipore). The Hoefer TE 22 Mini Tank Transphor Unit-System was used for blotting. Blots were then incubated with appropriate primary antibody (diluted in PBS containing 0.1% Tween 20, 5% BSA and 0.025% sodium azide) at 4°C overnight or for 2 h at room temperature. Subsequently, blots were incubated either with peroxidase-coupled secondary antibodies (diluted in 1% BSA in PBS-Tween 20) or with fluorescently labeled secondary antibodies (diluted in PBS-Tween 20 containing 5% BSA and 0.01% SDS) for 1 h at room temperature. Anti-mouse or anti-rabbit IgG, peroxidase-conjugated secondary antibody (Invitrogen) or fluorophore-coupled antibodies (anti-mouse or anti-rabbit IgG, coupled with Alexa 680 or Alexa 770, Invitrogen) were used. Detection of chemiluminescence or fluorescence was done with ECL films, Chemostar Imager (INTAS) or Odyssey Infrared Imaging system 2.1 (Li-Cor™ Biosciences).

For quantification each sample was loaded three times. Intensities of immuno-signals were quantified using Image J (NIH) software. All statistical analyses were performed with Prism 5 software (GraphPad Software) using unpaired t-test.

### Immunocytochemistry and fluorescence imaging

Cultured neurons were fixed with 4% formaldehyde and 4% sucrose in PBS for 10 min at room temperature. The cells were then washed, permeabilized and blocked with solution containing 10% FCS, 0.1% glycine and 0.3% Triton X-100 in PBS for 1 h. Both primary and secondary antibodies were diluted in PBS containing 3% FCS and applied for 1 h at room temperature. Secondary antibodies used were raised in donkey and coupled with Alexa 488 (Invitrogen), Cy3 or Cy5 (Jackson ImmunoResearch). Coverslips were mounted on microscopic slides with Mowiol (Calbiochem). The labeled neurons were examined using a 63× and 20× objective on a Zeiss Axio Imager A2 microscope equipped with Cool Snap EZ camera (Visitron Systems). The region of interest was set to nuclei using DAPI staining as mask. Mean gray values from nuclei of 40 inhibitory or excitatory neurons were measured using Image J (NIH). Values were obtained from two independent experiments and all statistical analysis were performed with Prism 5 software (GraphPad Software) using unpaired t-test. The brightness and contrast levels of the presented images were minimally adjusted (using Adobe Photoshop 5.0 software). No additional digital image processing was performed.

### Immunohistochemistry and confocal microscopy

Adult C57/BL6 mice were anesthetized with isoflurane and then transcardially perfused with PBS followed by fixative containing 4% paraformaldehyde (PFA) in PBS, pH 7.4. The brains were removed from the skull, post-fixed in the same fixative overnight at 4°C, cryoprotected by incubation with 0.5 M and 1 M sucrose, frozen with cold isopentane (precooled at −74°C) and stored at −20°C. Free-floating 30–40 µm thick sagittal brain sections were cut at the level of dorsal hippocampus using cryotome, washed with PBS, incubated with 1% Na-borhydride in PBS (to block aldehyl groups from PFA) and washed with PBS again. The slices were then blocked and permeabilized in 10% normal goat serum/0.3% Triton X-100 in PBS for 60 min and incubated overnight at 4°C on a shaker with primary antibodies diluted in the same blocking solution with 0.01% Na-azide. After washing with PBS, brain sections were blocked again with 0.4% BSA/0.3% Triton X-100 in PBS for 60 min followed by overnight incubation with appropriate secondary antibodies diluted in the same blocking solution. Cy3-conjugated donkey anti-mouse and donkey anti-rabbit (Jackson ImmunoResearch) and Alexa 488-conjugated donkey anti-rabbit (Invitrogen) secondary antibodies were used. Slices were then washed with PBS and mounted onto microscopic glass slides (Menzel, Germany) using DAPI-containing Vectashield (Vector Labs).

Images were taken with a Leica SP5 confocal microscope using 63× oil immersion objective with or without 4-fold zoom of scanner head and LCS software (Leica, Wetzlar, Germany). The brightness and contrast levels of the presented images were minimally adjusted using Image J software. No additional digital image processing was performed.

### Plasmid constructs

pEGFP-CtBP1 was generated by in frame insertion of cDNA of CtBP1-S (aa1–43) into pEGFP-C1 vector (Clontech). CtBP2 and CtBP2-S were amplified out of a pACT2 rat brain cDNA library (Clontech; Rat brain MATCHMAKER cDNA library; catalog number RL4005AH) using following primer sequences: CtBP2 forward 5′-aagactcgagatggcccttgtggataag-3′; CtBP2-S forward 5′-aagactcgagatgaacggccccct-3′, CtBP2 and CtBP2-S reverse 5′-tctgggtaccctattgctcgttggggtg-3′. The PCR products are XhoI/KpnI cloned into pEGFP-CW3 (derived from pEGFP-C2 (Clontech) by BglII digestion and religation to generate a C3 reading frame).

## Results

### Specificity of antibodies against CtBP1 and CtBP2

To prove the specificity of commercially available mouse and rabbit antibodies against both CtBP1 and CtBP2 we tested them on rat brain and retina homogenates and on cell lysates prepared from HEK293T cells expressing recombinant EGFP (enhanced green fluorescent protein)-CtBP1 and EGFP-CtBP2. The expression of both constructs in HEK293T cells was confirmed using EGFP specific antibody (Fig. 1D). The expression of retina specific CtBP2 variant RIBEYE was further confirmed by detection with a RIBEYE-specific antibody (Fig. 1G). Both mouse and rabbit antibodies against CtBP1 recognized clearly ubiquitously expressed CtBP1 migrating at 45 kDa in all samples, as well as the over-expressed EGFP-CtBP1 migrating at 75 kDa (Fig. 1B, C). They did not cross-react with the over-expressed EGFP-CtBP2 or with RIBEYE, the 120 kDa retina-specific product of the CtBP2 gene [5], confirming specificity of both antibodies for CtBP1. Both mouse and rabbit antibodies against CtBP2 recognized in lysates of transfected HEK293T cells two bands at about 40 and 45 kDa (Fig. 1E, F) corresponding to previously described short and long isoforms of CtBP2 [8]. In the retina homogenate both antibodies against CtBP2 recognized a double band of RIBEYE. Additionally, both CtBP2 antibodies also recognized over-expressed EGFP-CtBP2 fusion construct, but did not show cross-reactivity with EGFP-CtBP1, confirming their specificity for CtBP2 gene products. These data show that all CtBP antibodies used in this study are specific and thus suitable for investigation of the localization of CtBP isoforms in brain slices by immunostaining.

### Expression pattern of CtBP1 and CtBP2 in the adult mouse brain

**Figure 2:**
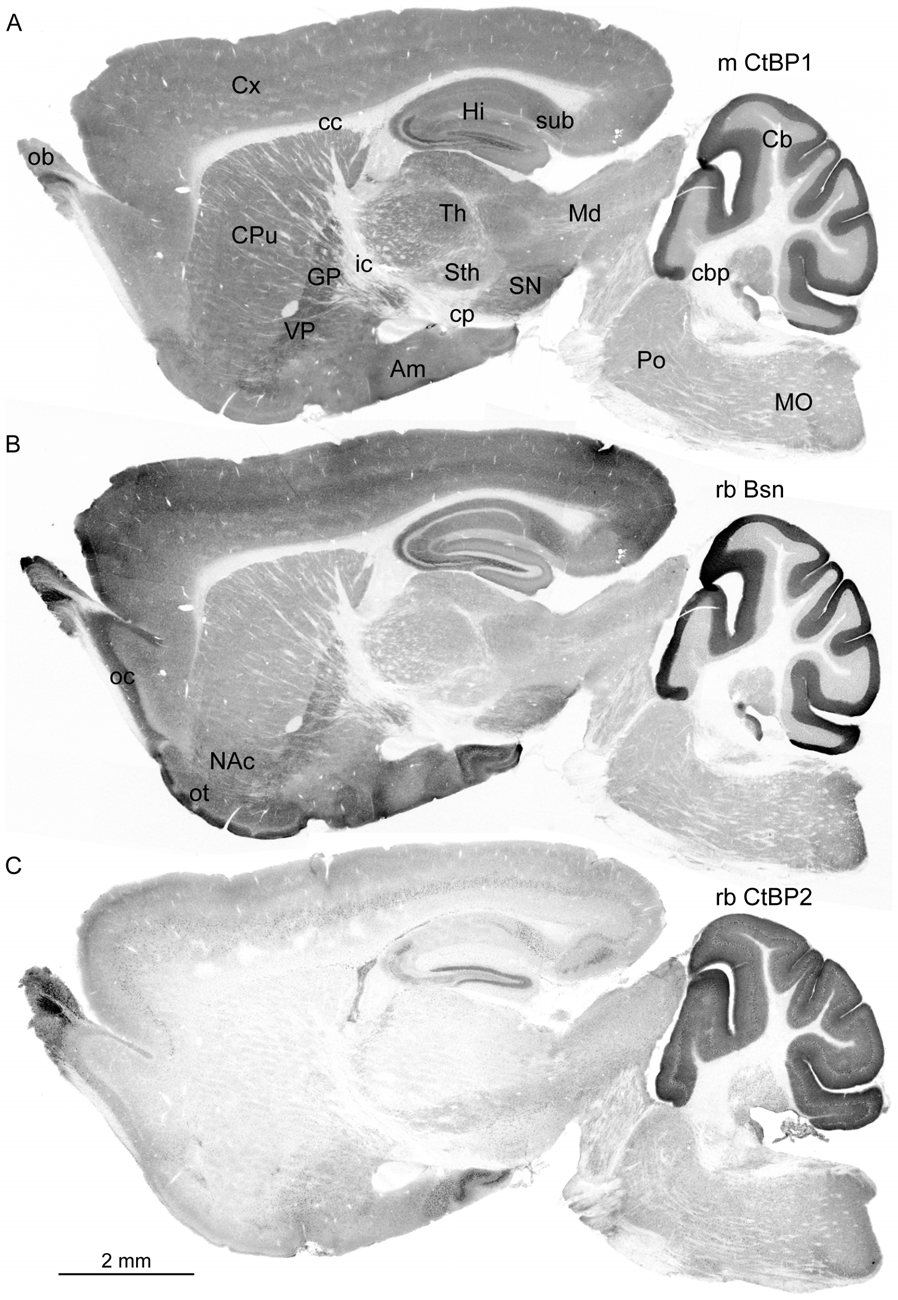
Overall expression pattern of CtBP1, Bassoon and CtBP2. The sagittal slices of adult mouse brain were stained with antibody from mouse against CtBP1 (A), from rabbit against Bassoon (Bsn) (B) and rabbit against CtBP2 (C) and corresponding fluorescently coupled secondary antibodies. A and B show staining with two antibodies in the same slice. Am – amygdala, Cb – cerebellum, cbp – cerebellar peduncle, cc – corpus callosum, cp – cerebral peduncle, CPu – caudate putamen, Cx – cortex, GP – globus pallidus, Hi – hippocampus, ic – internal capsula, Md – midbrain, MO – medula oblongata, NAc – nucleus accumbens, ob – olfactory bulb, oc – olfactory cortex, ot – olfactory tuberculus, Po – pons, SN – substantia nigra, Sth – subthalamus, sub – subiculum, Th – thalamus, VP – ventral palidium.

To investigate expression patterns of both CtBP family members we performed immunostaining with mouse antibody against CtBP1 and rabbit antibody against CtBP2 on fixed sagittal sections (lateral 1.8 mm in Fig. 2 and S1A, and 0.5 mm in Fig. S1B) of adult mouse brains. The specificity of used antibodies in the immunohistochemical staining was confirmed by staining with independently raised antibodies from rabbit against CtBP1 and from mouse against CtBP2, which resulted in identical staining pattern (Fig. S1). CtBP1 was previously described as nuclear and synaptic protein [18], [22]. To assess the dual synapto-nuclear localization the slices were co-stained with antibody against the presynaptic active zone marker Bassoon and the nuclear marker 4′,6-diamidino-2-phenylindole (DAPI). In a first step, we acquired overview images of whole slices using wide-field fluorescence microscopy and analyzed expression of both proteins throughout the brain.

**Figure 3:**
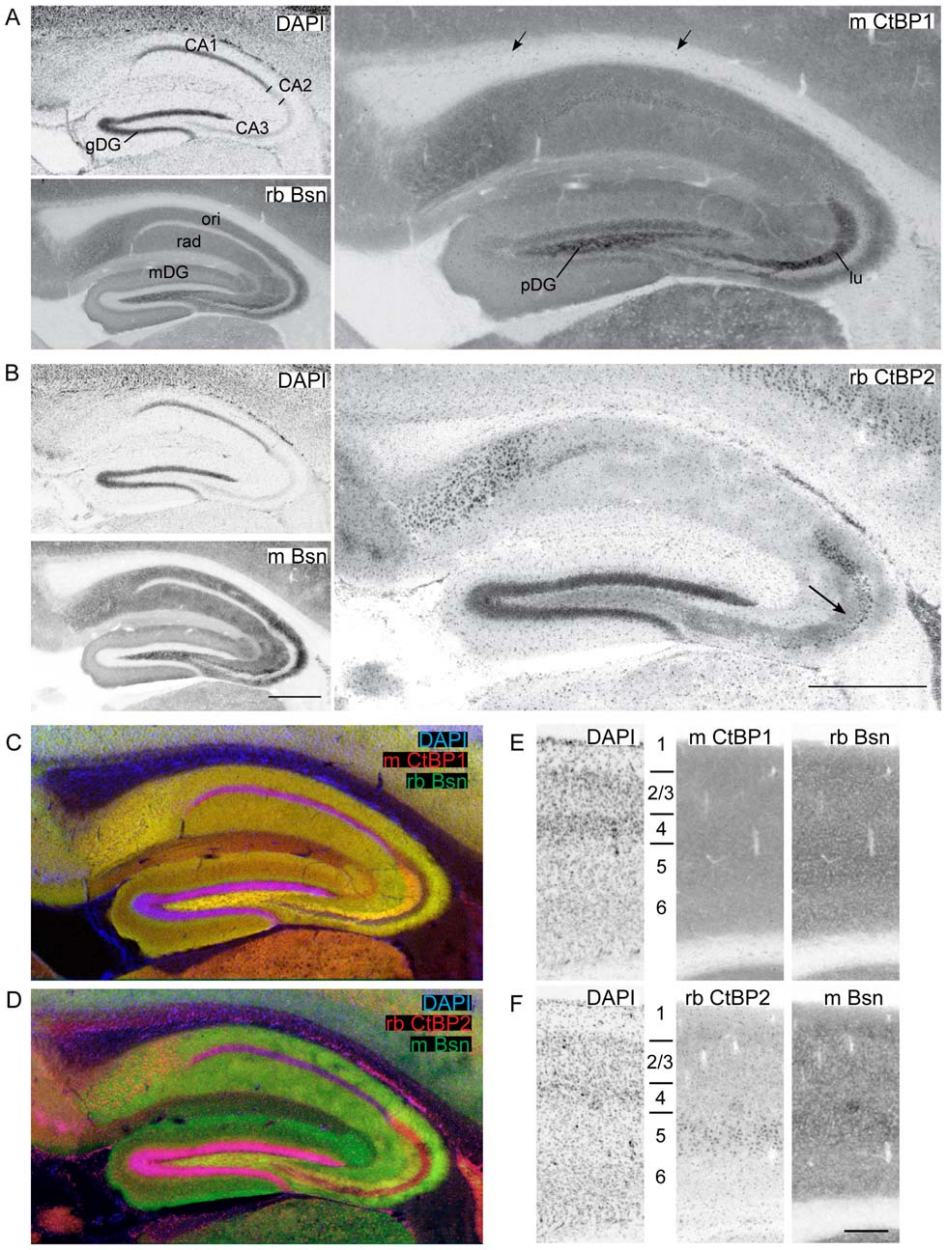
Expression of CtBP1 and CtBP2 in hippocampus and cortex. Images showing the region of hippocampus (A–D) and visual cortex (E, F) taken from sagittal mouse brain slices stained with DAPI, antibodies against CtBP1 or 2 and Bassoon and corresponding fluorescent secondary antibodies. The images in A, B, E, F always show staining of the same slice. In C and D overlay of staining in all channels are shown. The bars are 600 µm in A and B, and 250 µm in E, F. Arrows in A show scattered cell bodies labeled with mCtBP1 antibody in corpus calosum, arrow in B depicts immunoreactivity of rbCtBP2 in the stratum lucidum. CA1–3 – CA1 to 3 regions of hippocampus, gDG – granular layer of dentate gyrus, lu- stratum lucidum, mDG – molecular layer of dentate gyrus, ori – stratum oriens, pDG – polymorph dentate gyrus, rad – stratum radiatum.

**Overall expression:** CtBP1 expression was visible throughout the brain (Fig. 2A) and, in addition to nuclear staining, mirrored well the distribution of synaptic marker protein Bassoon in the brain neuropil regions (Fig. 2B). This is well compatible with the synaptic localization of CtBP1, as it was reported in dissociated neuronal cultures from hippocampus earlier [18]. Strong immunoreactivity for CtBP1 was detected in forebrain and cerebellum, whereas it was lower in brainstem (i.e. medulla oblongata, pons and midbrain) with the exception of strong expression observed in the substantia nigra. The white matter of the brain e.g. corpus callosum, internal capsule, cerebral and cerebellar peduncles and tract of trigeminal nerve, contained only few scattered cell bodies with CtBP1 immunoreactivity (Fig. 2A and arrows in Fig. 3A). In the diencephalon, the dorsal thalamus shows stronger expression than the subthalamus. All telencephalic structures displayed a clear labeling. From basal nuclei the globus pallidus and the ventral pallidum showed more CtBP1 immunoreactivity than caudate putamen and ventral striatum. In contrast to this ventral striatum (i.e. nucleus accumbens and olfactory tubercle) exhibited particularly high expression of protein Bassoon (Fig. 2B).

The immunoreactivity for CtBP2 was highest in olfactory bulbs and in cerebellum (Fig. 2C), where in contrast to CtBP1 and Bassoon also layers containing cell bodies displayed strong staining. Noticeable staining of cell bodies throughout the brain could be observed, likely corresponding to previously described nuclear expression of this protein in the brain [18]. Unexpectedly, diffuse immunoreactivity was observed in neuropil layers of hippocampus (arrow in Fig. 3B) and cerebral cortex (Fig. 2C) suggesting synaptic localization of CtBP2.

**Hippocampal formation (** **Fig. 3A–D** **) and cerebral cortex (** **Fig. 3E, F** **):** In the hippocampal formation CtBP1 expression was detectable in all neuropil layers. It was high in subiculum and especially strong in polymorphic layer of dentate gyrus and in the stratum lucidum of the hippocampal CA3 region. Overall, labeling of hippocampal neuropil layers for CtBP1 resembled closely that of Bassoon (**Fig. 3C**). In contrast to Bassoon, CtBP1 could also be detected in the granule cell layer of dentate gyrus, in the pyramidal cell layer of CA1 and weaker also in CA2, whereas there was only very weak staining in the pyramidal cell layer of CA3 region (Fig. 3A). In the cerebral cortex the staining for CtBP1 was detectable in all layers (Fig. 3E).

CtBP2 immunoreactivity was strongest in the cell body layers of dentate gyrus, CA3 and especially in CA2, with no detectable immunoreactivity in the CA1 pyramidal cell layer (Fig. 3B). Cells with strong immunoreactivity were also found in subiculum and all neuropil layers of the hippocampus. Strikingly, diffuse staining was evident in neuropil layers of CA1–3 and was especially strong in the stratum lucidum of CA3 and in the polymorphic cell layer of dentate gyrus pointing towards synaptic localization of CtBP2 in hippocampus (arrow in Fig. 3B). In the cerebral cortex, remarkably strong staining was found in cells in upper layer 5 although scattered cells stained for CtBP2 could be found in all cerebral layers. Considerable neuropil staining was found in layers 1–4 and also in upper layer 5 (Fig. 3F).

**Figure 4:**
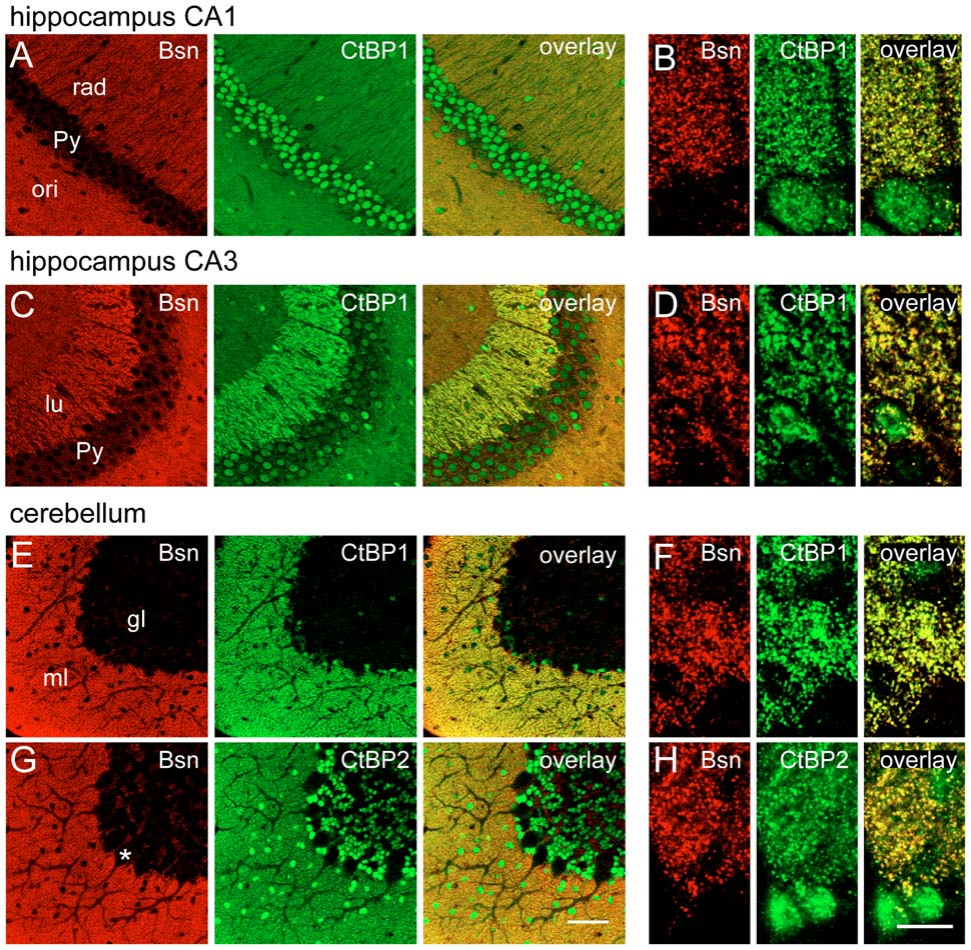
Confocal images of CtBP1 and CtBP2 localization at synapses of hippocampus and cerebellum. Slices were stained with following antibodies: anti CtBP1 from mouse and anti Bsn from rabbit (A–F) and anti CtBP2 from rabbit and anti Bsn from mouse in G and H. Images from hippocampal region CA1 (A, B), and CA3 (C, D) and from cerebellum (E–H) are shown. B shows a high-resolution scan of transition between pyramidal cell layer and stratum radiatum, D transition between pyramidal cell layer and stratum lucidum and F and H transition between granular and molecular cell layer. Note significant overlap of staining for CtBPs and Bassoon in punctate pattern in neuropil in high-resolution scans. Scalebars are 50 µm in G and 10 µm in H. gl- granular layer, lu – stratum lucidum, ml – molecular layer, ori – stratum oriens, Py – pyramidal cell layer, rad – stratum radiatum, the cell body of a Purkinje cell is marked by asterisk.

**Cerebellum:** In cerebellum staining of CtBP1 was restricted to the neuropil of the molecular layer suggesting its synaptic localization (Fig. 2A, see also Fig. 4E). In contrast to that diffuse CtBP2 immunoreactivity was present in both molecular and granular layers (Fig. 2C, see also Fig. 4G). Cells showing considerable CtBP2 immunoreactivity were scattered within molecular and granular layers.

### Subcellular localization of CtBPs in brain neurons

The overall expression pattern of CtBPs in brain suggested their expression in neuropil and neuronal cell bodies, which might reflect their dual synaptic and nuclear localization. To support this conclusion we acquired high-resolution confocal images allowing precise assignment of immunoreactivty to subcellular structures. Co-staining with a specific antibody against Bassoon was used to mark presynaptic active zones (Fig. 4). Whereas most nuclei of CA1 granular cell layer displayed strong CtBP1 staining only few nuclei expressed weaker CtBP1 staining in the CA3 region (Fig. 4A, C). The high-resolution images revealed very good co-localization of CtBP1 staining with staining for Bassoon in the stratum radiatum of CA1 and the stratum lucidum of CA3 confirming localization of CtBP1 to hippocampal synapses formed by Schaffer collaterals to CA1 pyramidal cells and by mossy fibers on CA3 pyramidal cells (Fig. 4B, D). Likely due to the low signal to noise ratio in the high-resolution images of staining for CtBP2 in neuropil of hippocampus, the synaptic staining could not be clearly detected. Only labeling of cell nuclei scattered through all layers of hippocampus (also visible in Fig. 3B and D) could be detected at this level.

In cerebellum only weak nuclear staining was detected for CtBP1 in cells throughout the molecular and granular cell layers (Fig. 4E). The inspection of high magnification images revealed a high degree of overlap of staining for Bassoon and CtBP1, confirming synaptic expression of CtBP1 in cerebellum (Fig. 4F). Considerable immunoreactivity for CtBP2 was evident in nuclei of cells of granular and molecular cell layer (Fig. 4G). Interestingly, the nuclei of Purkinje cells were free of CtBP2 staining. High resolution images revealed punctate staining for CtBP2 in the neuropil of the cerebellar molecular layer, which overlapped very well with the staining for synaptic marker Bassoon, strongly suggesting synaptic expression of CtBP2 in cerebellum (Fig. 4H).

### Expression of CtBP1 and CtBP2 in inhibitory and excitatory neurons

**Figure 5:**
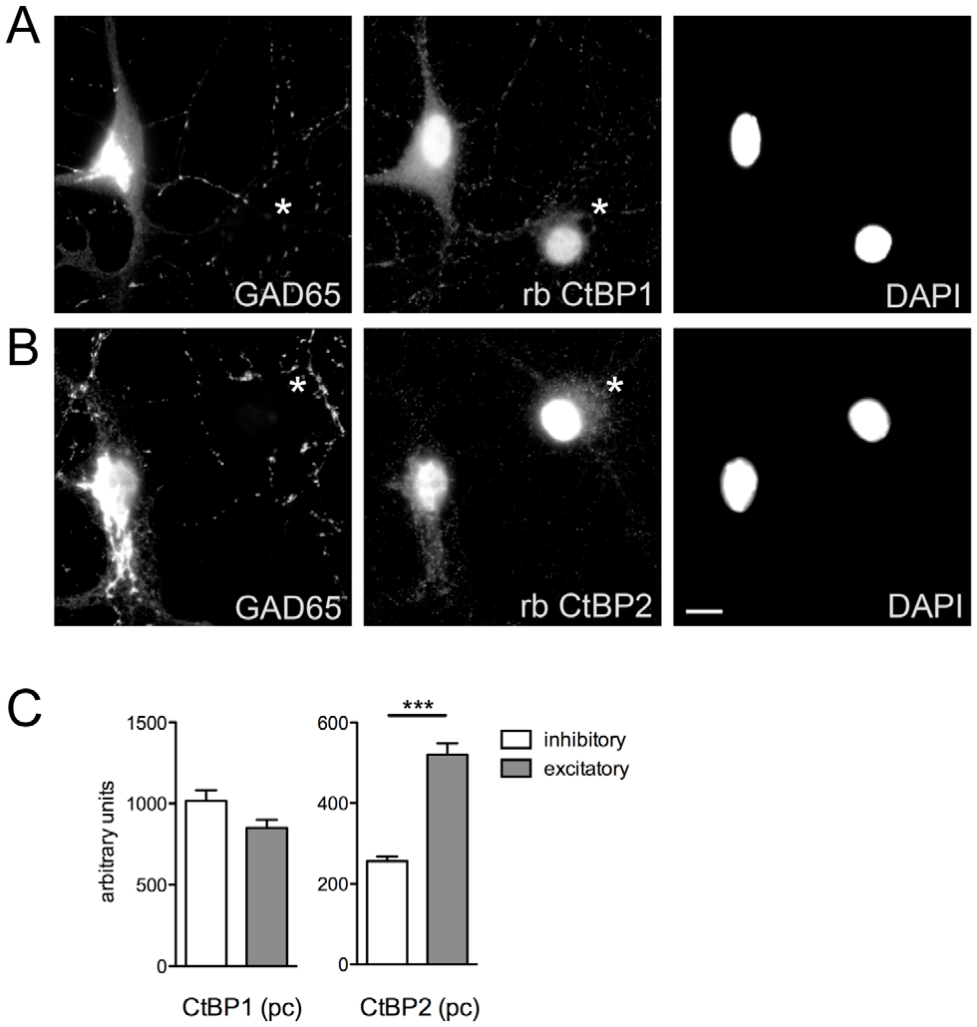
Cell-type specific expression of CtBP1 and CtBP2. Rat hippocampal neurons grown for 14 days in dissociated cultures were stained with rabbit antibodies against CtBP1 (A) or CtBP2 (B). Staining with GAD65-specific antibody was used to mark cell bodies of inhibitory neurons. Non-stained neurons were considered to be excitatory (marked by asterisk). The CtBP1 and CtBP2 immunoreactivity in nuclei (highlighted by staining with DAPI) was measured and quantified. The quantification revealed significantly lower nuclear expression of CtBP2 in GAD65-positive neurons compared with GAD65 negative ones, whereas CtBP1 expression was not different in these two cell types. Scalebar is 10 µm.

The immunoreactivity distribution in cell nuclei of hippocampus was remarkably different for CtBP1 and CtBP2. To test whether the specificity of excitatory or inhibitory neurons is the factor determining the level of nuclear expression of both proteins we stained hippocampal neurons grown for 14 days in dissociated cultures and quantitatively analyzed nuclear staining for both CtBP1 and CtBP2. To distinguish between inhibitory and excitatory neurons we used a marker of inhibitory neurons GAD65, which labeled about 25% of cells in our cultures. The cell nuclei were labeled with DAPI. Immunoreactivity for CtBP1 and CtBP2 in cell nuclei was quantified and compared between excitatory and inhibitory cells. There was only slightly higher nuclear expression of CtPB1 in inhibitory cells compared to excitatory ones (inhibitory 1016±66.16 vs. excitatory 852.0±48.60 arbitrary units, mean±SEM, N = 40, P = 0.048; t-test; Fig. 5A, C). In contrast, the immunoreactivity for CtBP2 was about twice as high in excitatory cells compared to inhibitory ones (inhibitory 256.2±12.07 vs. excitatory 520.7±27.53, N = 40, P<0.0001, t-test; Fig. 5B, C).

### Proof of synaptic localization of CtBP1 and CtBP2 by immunoelectron microscopy

**Figure 6:**
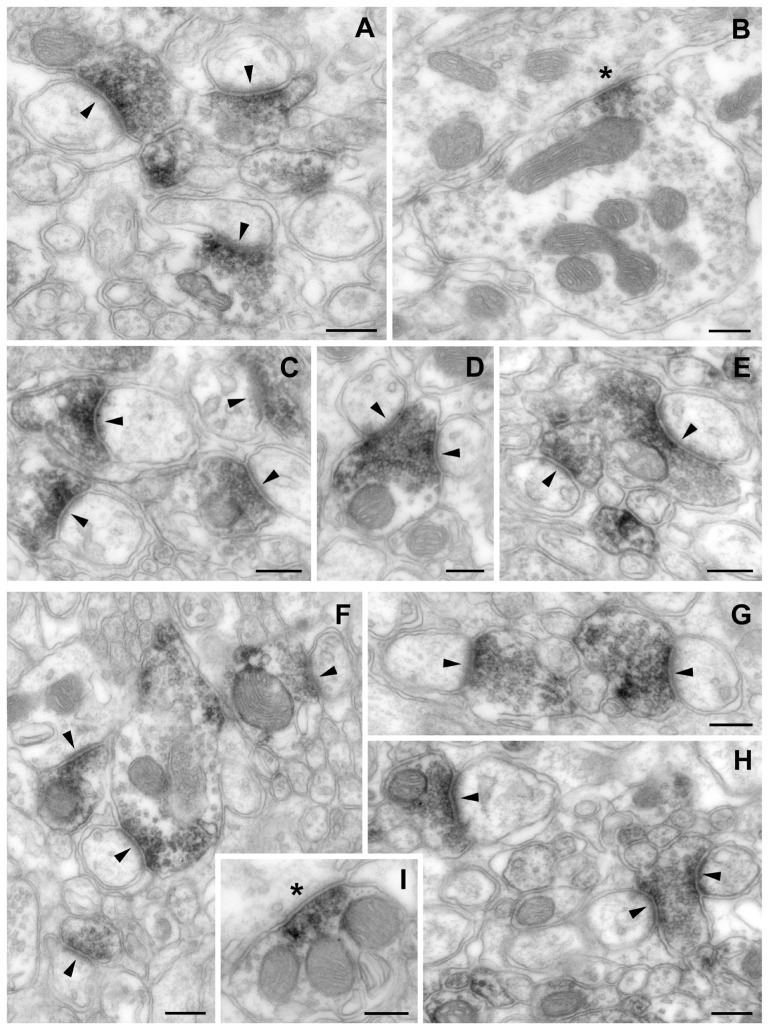
Localization of CtBP1 and CtBP2 in the molecular layer of the cerebellum. Electron micrographs showing immunoreactivity for CtBP1 (A–E) and CtBP2 (F–I) in presynaptic elements in the molecular layer of the cerebellum as detected by pre-embedding immunoperoxidase method. Arrowheads mark the postsynaptic density of clearly displayed asymmetric synapses and asterisks mark the postsynaptic side of symmetric synapses. (A) and (C) show examples of immunolabeled axonal varicosities (of presumed parallel fibers) contacting unstained postsynaptic elements (thorns of Purkinje dendrites). (B) Symmetric synaptic junction between a CtBP1-immunopositive presynaptic element (presumed basket cell axon) and a Purkinje cell body. The peroxidase reaction product is concentrated at the synaptic contact zone. (D, E) Axon varicosities synapse two dendritic thorns at the section plane. In D the densely packed vesicles co-localize with the peroxidase reaction product. In E the immunoreactivity is concentrated at region apposing PSD, however, an axonal profile without synaptic contact at the section plane shows also a strong labeling.(F–H) Immunopositive varicosities make asymmetric contacts to thorns and a dendrite (in the left of F). A gradient of increasing amount of peroxidase reaction product towards the synaptic contact zone is detectable. Postsynaptic structures are free of immunoreactivity. In H some axonal profiles are also labeled. (I) CtBP2-immunolocalization in a presumed basket cell axon making a symmetric contact on a Purkinje cell body. The peroxidase reaction product is accumulated in the presynaptic element. Scale bars correspond to 250 nm.

To confirm the synaptic localization of CtBP1 and CtBP2 we analysed the molecular layer of the cerebellar cortex applying pre-embedding immunoperoxidase technique. We found populations of clearly CtBP1- (Fig. 6A–E) as well as CtBP2-immunopositive (Fig. 6F–I) synapses scattered throughout the molecular layer. In all cases, the presynaptic element was labeled and the postsynaptic part was free of staining. The majority of synaptic contacts made by immunopositive presynaptic elements are asymmetric (Fig. 6A, C–H). The reaction product in the presynaptic compartments showed a gradient of increasing amount toward the synaptic cleft. However, sometimes also immunopositive axon profiles (e.g.parallel fiber axons) can be observed in the case of CtBP1 and more frequently in the case of CtBP2. Also in presynaptic profiles, which form symmetric contacts, CtBP1 and CtBP2 immunoreactivity could be detected near the synaptic contact zone (Fig. 6B, I).

### Analysis of expression of CtBPs in mouse brain by immunoblotting

**Figure 7:**
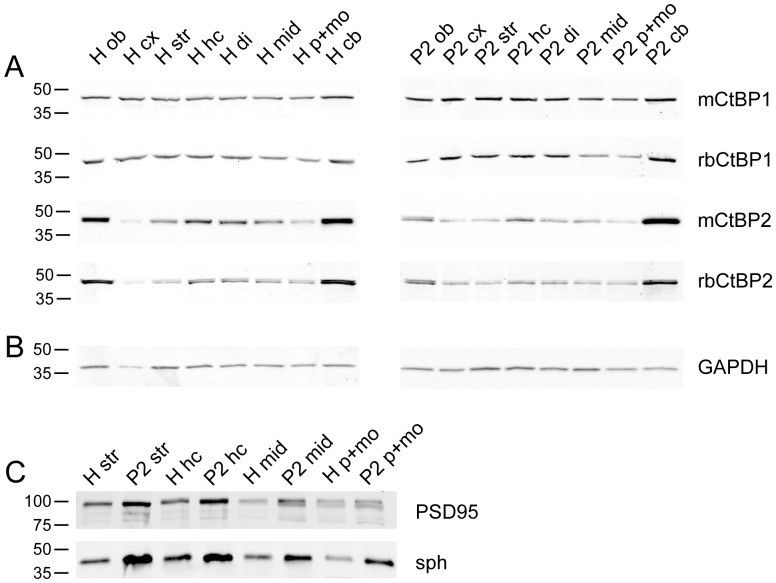
Expression of CtBP1 and CtBP2 in different brain regions. Equal amounts of homogenates (H) and P2 fractions (P2) from olfactory bulbs (ob), cortex (cx), striatum (str), hippocampus (hc), diencephalon (di), midbrain (mid), pons with medulla oblongata (p+mo) and cerebellum (cb) isolated from brains of adult mice were analysed on immunoblots using antibodies from mouse and rabbit against CtBP1 and CtBP2 (A). In all experiments immunodetection of GAPDH was used to control for loading of equal amounts of protein (B) and immunodetection of synaptophysin and PSD95 to control for successful enrichment of membrane-associated brain proteins in P2 fraction (C). Note higher expression of CtBP2 in olfactory bulbs and cerebellum in both homogenates and P2 fraction. Bars and numbers indicate position and size (in kDa) of the molecular weight markers.

To assess relative expression levels of CtBPs throughout the brain, we isolated olfactory bulbs, cortex, striatum, hippocampus, diencephalon, midbrain, pons with medulla oblongata and cerebellum from brains of adult mice. The tissue samples were homogenized and post-nuclear crude membrane fraction P2 was prepared by differential centrifugation. Same protein amounts (10 µg) of homogenates and P2 fraction containing samples were separated on SDS-gels and blotted prior to immunodetection with specific antibodies against CtBP1 and CtBP2 (Fig. 7A). The immunoreactivity for GAPDH was detected to control for quality of samples and equal loading (Fig. 7B). The generally higher immunoreactivity for synaptic vesicle protein synaptophysin and postsynaptic density marker PSD95 in P2 compared to homogenates confirmed successful enrichment of synaptic proteins in the P2 fraction (Fig. 7C). Specific antibodies from mouse and rabbit were used for immunodetection of CtBP1 and CtBP2 giving equivalent results. The expression of CtBP1 in homogenates and P2 fractions was the same throughout all isolated brain regions. However, much higher levels of CtBP2 were detected in both homogenates and P2 fractions from olfactory bulbs and cerebellum, which is in good agreement with results of immunostaining shown in Fig. 2 and S1 and further discussed below (see section “Expression pattern of CtBP1 and CtBP2 in the adult mouse brain”).

**Figure 8:**
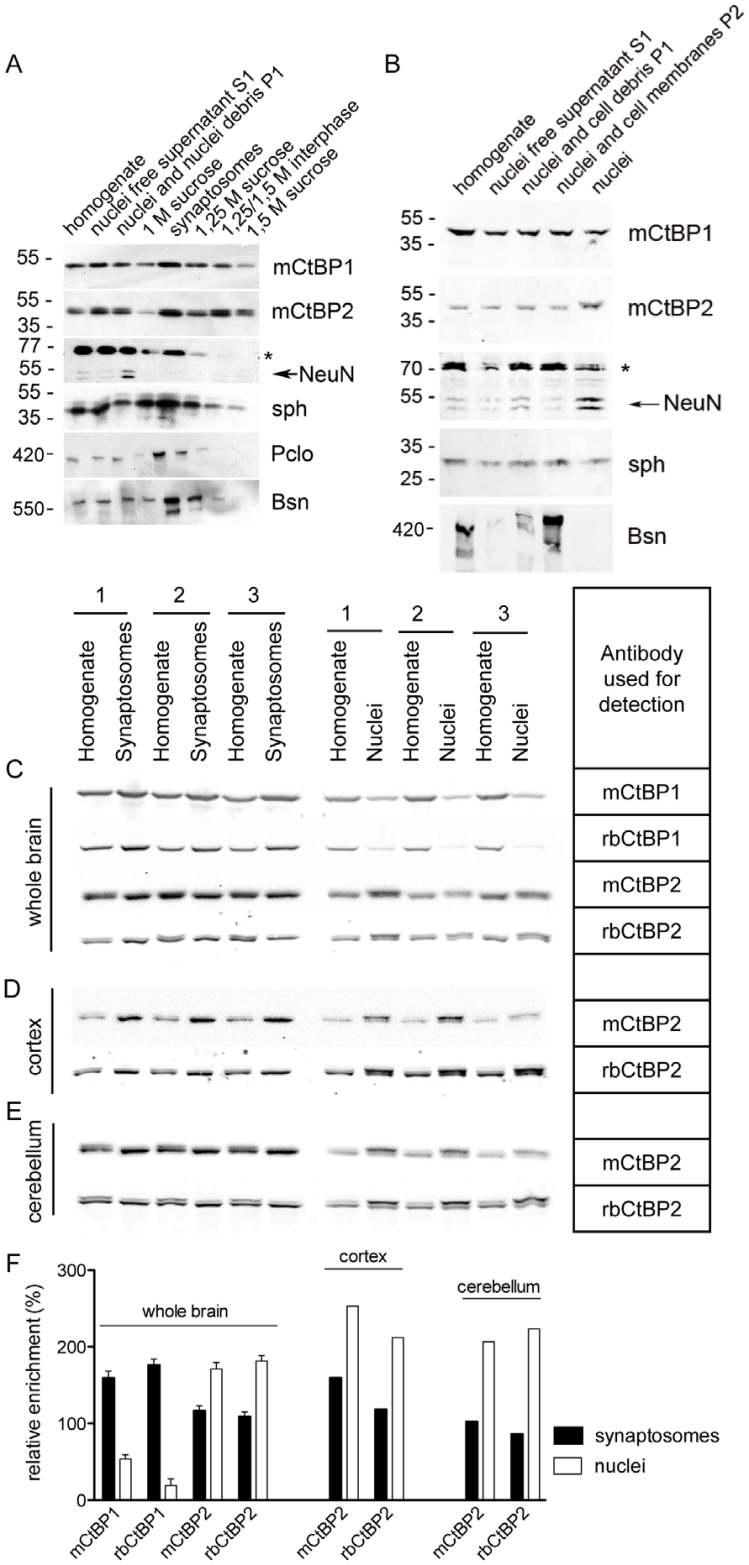
Quantitative analysis of nuclear and synaptosomal expression of CtBP1 and CtBP2. Synaptosomal (A) and nuclear (B) fraction from whole rat brain were analysed with antibodies against CtBP1, CtBP2, nuclear marker NeuN and synaptic markers synaptophysin (sph), Bassoon (Bsn) and Piccolo (Pclo). Bars and numbers indicate position and size (in kDa) of the molecular weight markers. The arrow shows specific double-band corresponding to NeuN, the band migrating above 70 kDa marker is not specific. Note enrichment of nuclear marker NeuN in nuclear fraction and its absence in synaptosomes. Sph, Bsn and Pclo are enriched in synaptosomes. Expression levels of CtBP1 and CtBP2 in nuclear and synaptosomal fraction prepared from whole brain (C), cortex (D) or cerebellum (E) were detected using specific antibodies from mouse and rabbit. The enrichment of signal in the synaptosomal or nuclear fraction was expressed in percentages relative to signal measured in homogenates. Note higher relative expression in synaptosomes than in nuclei for CtBP1 in contrast to higher relative expression of CtBP2 in nuclei compared to synaptosomes. The plot in F shows the results of quantification; bars represent the mean values, whiskers the SEMs.

The analysis of immunostaining in brain slices suggested that both CtBP1 and CtBP2 are localized in nuclei and synapses. To investigate quantitatively the dual synapto-nuclear distribution of these proteins we prepared synaptosomes and nuclei from fresh whole adult mouse brains. The quality of synaptosomal and nuclear preparations was controlled by detection of nuclear (NeuN) and synaptic (synaptophysin, Bassoon and Piccolo) marker proteins in the all fractions derived from both protocols (Fig. 8A, B). We loaded the same amount of probes on SDS-gels, performed quantitative immunoblots and measured relative enrichment of CtBP1 and CtBP2 in both fractions respective to brain homogenate, which was the starting material for the both preparations (Fig. 8C). This analysis showed that CtBP1 is much more enriched in synaptic fractions as compared to nuclei. In contrast, CtBP2 was preferentially associated with nuclei compared to synapses in samples prepared from whole brains (Fig. 8C). The staining of brain slices with antibody against CtBP2 revealed strong immunoreactivity in neuropil of cerebellum but much weaker staining of neuropil of cortex, where, in contrast, strong nuclear staining of some neurons was evident (Fig. 2C). We asked whether the strong synaptic localization of CtBP2 in cerebellum is due to preferential sorting of this protein to synaptosomal compartment in this brain region. To approach this question we prepared synaptosomal and nuclear fractions from cortices and cerebella of adult mice and compared relative synapto-nuclear distribution of CtBP2 in both regions (Fig. 8D, E). The quantitative analysis revealed comparable synapto-nuclear distribution in cortex and cerebella (Fig. 8F). Thus, the low marking of neuropil in cortex is due to low expression of CtBP2 in this region rather than to lack of its targeting to synapses.

### CtBP2-S lacking NLS is the predominant synaptic isoform

**Figure 9:**
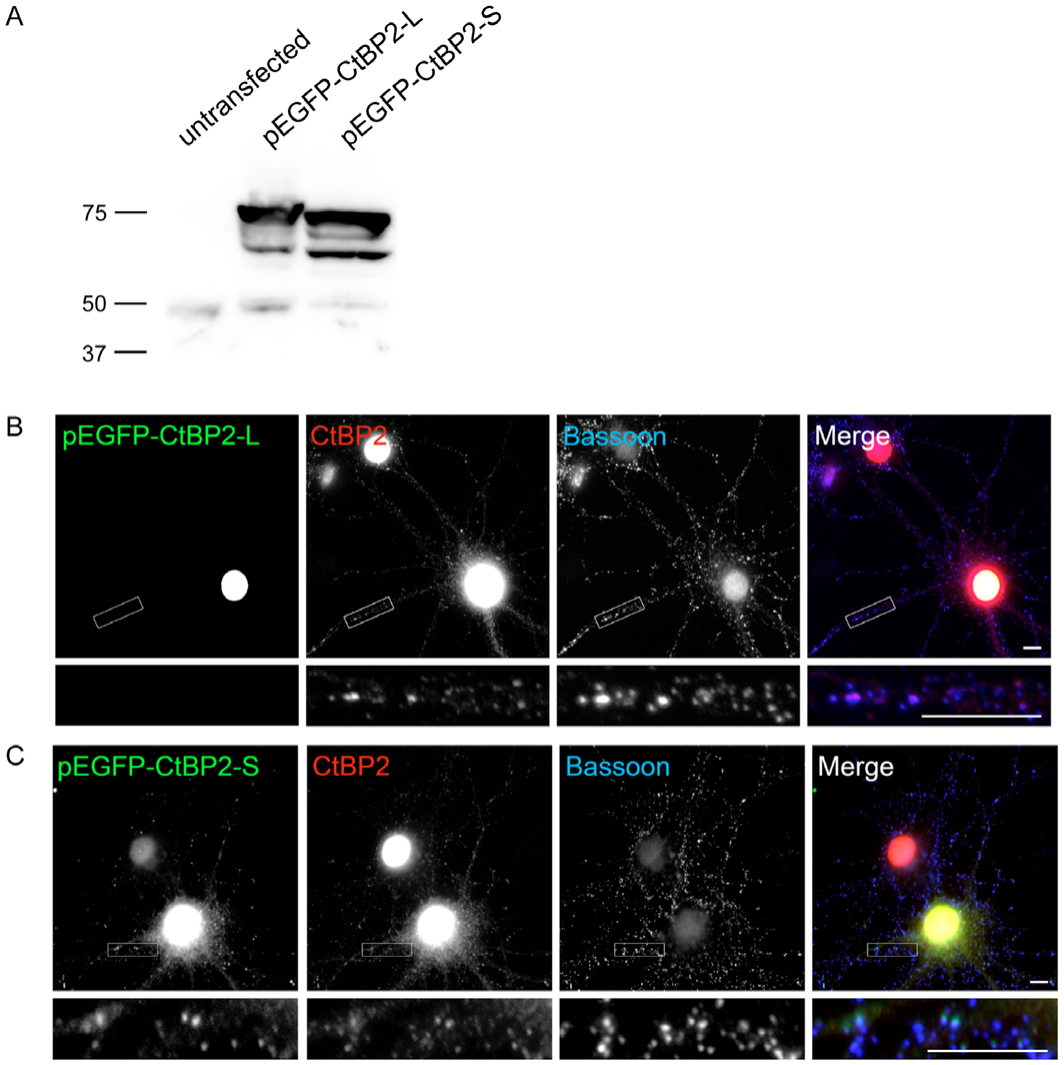
Differential subcellular localization of CtBP2-L and CtBP2-S isoforms in neurons. EGFP-tagged CtBP2-L and CtBP2-S fusion proteins could be detected using mouse antibody against CtBP2 in the lysates from HEK293T cells transfected with both expression constructs (A). Bars and numbers in C indicate position and size (in kDa) of the molecular weight markers. The highest bands represent the full-length fusion proteins, the weaker bands of lower apparent Mw correspond to degradation products. The EGFP-tagged CtBP2-L (B) and CtBP2-S (C) were expressed in rat hippocampal neurons and their synapto-nuclear localization was assessed by fluorescence microscopy. Staining with rabbit antibody against Bassoon was used to identify synapses. The CtBP2-L can be detected predominantly in the nuclei of transfected cells, while the expressed CtBP2-S localizes to nuclear and synaptic compartment. The higher magnification micrographs correspond to the boxed region of overview images and show synaptic localization of endogenous CtBP2 stained with mouse antibody against CtBP2 and of overexpressed EGFP-CtBP2-S, but not of EGFP-CtBP2-L. Scale bars are 5 µm in overview image and 10 µm in high-magnification image.

It has been proposed previously, that the presence of a nuclear localization signal (NLS) in one of the three CtBP2 splicing isoforms crucially determines its nuclear localization in cells [8]. The minimal difference between calculated Mw of CtBP2-L (containing the NLS; 48,9 KDa) and the one of CtBP2-S isoforms (lacking NLS; 46,2 kDa) makes it difficult to resolve these isoforms using normal ECL detection captured by film exposure or by CCD camera. Therefore, we established detection using fluorescent secondary antibody and signal detection by Odyssey scanning device. Using this technique we could detect two bands immunoreactive for CtBP2 in total brain homogenates (Figs. 7, 8). Interestingly, the lower band was specifically enriched in the synaptosomal fraction (Fig. 8), whereas the upper band was more abundant in nuclear fraction, which is in good agreement with the assumption that the NLS is the main determinant of nuclear targeting for CtBP2. Furthermore, we generated vectors driving expression of EGFP-tagged CtBP2-L and CtBP2-S fusion proteins in mammalian cells, which were first tested in HEK293T cells. We could successfully detect EGFP-CtBP2-L and EGFP-CtBP2-S fusion proteins in cell lysates of transfected cells using CtBP2 specific antibodies from mouse (Fig. 9A). To support our hypothesis that CtBP2-S is the main isoform localized to synaptic compartment in neurons, we expressed EGFP-CtBP2-L and EGFP-CtBP2-S in cultured hippocampal neurons and analyzed their subcellular localization. The EGFP-CtBP2-L was localized exclusively in nuclei of transfected neurons (Fig. 9B). In contrast to that, the EGFP-CtBP2-S was localized in both nuclei and in presynapses of transfected neurons (Fig. 9C). This observation strongly suggests that the NLS-lacking isoform CtBP2-S localizes in synapses and nuclei, whereas the NLS-containing isoform CtBP2-L is predominantly targeted to nuclear compartment of neurons.

## Discussion

### CtBP1 and CtBP2 proteins have different expression patterns in brain

In this study we present detailed appraisal of expression pattern of members of CtBP protein family, CtBP1 and CtBP2 in rodent brain. To this end we utilized antibodies specific to either CtBP1 or CtBP2. The antibodies did, however, recognize all isoforms known to date expressed from the individual genes. However, as the specific antibody to ribbon-specific CtBP2 variant RIBEYE did not show any signal in homogenates from whole brain (data not shown), the signals described here are most likely derived from the long and short isoforms of CtBP1 and CtBP2. Despite of the rather similar, widespread and even expression of mRNA for both CtBP1 and CtBP2 throughout the brain (see Allen Mouse Brain Atlas [Internet]. Seattle (WA): Allen Institute for Brain Science. ©2009. Available from: http://mouse.brain-map.org) we found distinct distribution patterns for their protein products. Specifically expression levels and subcellular localization varied in different brain regions for the two genes. Our biochemical analysis revealed large differences in the expression levels of CtBP2 in different brain regions with especially high expression in cerebellum and olfactory bulbs comparing to remaining areas. This was different from CtBP1, which was about evenly expressed in all tested brain regions. The distribution of immunoreactivity on brain slices obtained with specific antibodies against both proteins confirmed these findings. For several brain regions, we found nearly complementary expression of both members of the CtBP family; e.g. cell bodies of hippocampal pyramidal cell layer showed immunoreactivity for CtBP1 mainly in CA1 region and for CtBP2 in CA2 and CA3 regions. Cell nuclei of granule cells of dentate gyrus displayed, however, considerable labeling for both CtBP1 and CtBP2. Interestingly, stainings for CtBP1 and CtBP2 in glutamatergic and GABAergic cells in dissociated hippocampal cultures revealed that nuclear expression of CtBP2 is much higher in excitatory cells compared to inhibitory ones, whereas CtBP1 showed slightly higher levels in inhibitory cells. The nuclear transcriptional co-repressors CtBP1 and CtBP2 are highly related and functionally redundant. To date only few studies sought to identify exclusive roles for CtBP1 or CtBP2 [15], [23]. However, the individual expression patterns of these proteins in the brain reported here suggest that they might play important non-overlapping roles in the regulation of neuronal differentiation and/or in the cell-type specific function.

### CtBP2 is a newly detected synaptic protein

While CtBP1 showed widespread expression in brain neuropil, with rather low immunoreactivity in the cell bodies, CtBP2 was detected mainly in cell body-rich regions throughout the brain. This is in good agreement with the strong expression of CtBP2 in the somata of cultured hippocampal neurons reported previously [18]. Unexpectedly, we detected strong labeling of neuropil with CtBP2-specific antibodies in the cerebellum and to lower extent also in the mossy fiber pathway of hippocampal formation and in the cerebral cortex of immunostained brain slices. High-resolution confocal imaging showed unambiguously CtBP2 immunoreactivity co-localizing with the immunoreactivity for the presynaptic marker Bassoon in the molecular layer of the cerebellum suggesting a presynaptic localization of CtBP2 in this brain region. The localization of CtBP2 in presynaptic boutons was further confirmed by immunoelectron microscopy. Different to our previous study [18] and probably due to a higher affinity of antibodies used for staining here, we were able to detect an immunoreactivity of CtBP2 in the synapses of cultured neurons from hippocampus. Intriguingly, we observed a high level of overlap of CtBP1 and CtBP2 localization with the one of the active zone protein Bassoon in different brain regions. The immunoelectron microscopy detected both CtBPs in the close vicinity of presynaptic active zone, where Bassoon is exclusively located. In Bassoon-mutant mice the presynaptic ribbons are not correctly anchored to the presynaptic plasma membrane and float in the cytoplasm of photoreceptor or inner hair cells [12], [24]. We reported a physical interaction of Bassoon with the CtBPs earlier and postulated a role for this interaction in the anchoring of synaptic ribbons, which are supposed to be structured by the CtBP2 gene product RIBEYE [13], to active zones of retinal photoreceptors and inner hair cells of cochlea [18]. Whether the core scaffold of presynaptic cytomatrix Bassoon [25] also controls the synaptic localization of CtBPs at conventional synapse remains to be tested.

What are the functions of CtBPs at presynapse? The main function of presynaptic termini is the evoked release of neurotransmitter, which is based on precisely controlled sequence of membrane budding, trafficking and fusion steps [26]. CtBP1 was implied in the regulation of membrane fission, indispensable for membrane fusion and budding process [9], and might be therefore involved in the fine-tuning of presynaptic neurotransmitter release.

### Determinants controlling synapto-nuclear distribution of CtBP2

In this study we demonstrated that both CtBP1 and CtBP2 showed a dual synapto-nuclear localization in neuronal cells. Using cell fractionation protocols and quantitative immuno-Western blotting we have shown that CtBP1 is much more enriched in synaptosomal than in the nuclear fraction in contrast to CtBP2 which seems to be targeted more to nuclei. However, in cerebellum we observed strong immunoreactivity of CtBP2 in neuropil. Our quantitative analysis revealed that the strong immunoreactivity for CtBP2 in the synaptic layers of cerebellum is not due to its preferential synaptic targeting, but is rather due to the especially high expression of CtBP2 in this brain region. Our immunoblot analysis also revealed differential synapto-nuclear distribution of two previously described CtBP2 isoforms CtBP2-L and CtBP2-S. The NLS-lacking CtBP2-S was the main synaptic *CtBP2* gene product whereas the NLS-containing CtBP2-L was relatively enriched in the nuclear fraction. We could further confirm this result showing that the over-expressed CtBP2-S reaches synapses in contrast to over-expressed CtBP2-L, which was predominantly targeted to the nuclei of transfected neurons. Therefore, we suggest that the NLS is the main determinant assuring the sorting of CtBP2-L into nucleus. This is further supported by observation that the CtBP1 chimera construct containing the NLS of CtBP2 [8] was not targeted to synapses, but accumulated in cell nuclei when expressed in neurons (AF unpublished data). Although CtBP2-S was described as cytoplasmic protein in non-neuronal cells previously [8], its function remained elusive. Specific regulation of gene expression is required in the processes of neuronal plasticity, which is in turn fundamental for complex brain function including learning and memory formation [27]. However, the mechanisms how synapses communicate with the neuronal cell nuclei are still not well understood and involvement of specific synapto-nuclear messengers was hypothesized [28], [29]. CtBPs were shown to shuttle between nucleus and cytoplasm in non-neuronal cells [8] and were implied in the repression of transcription induced by Wnt and TGF-β/BMP signalling pathways [30], [31], which also plays pivotal roles during synaptogenesis and synaptic plasticity in neurons [32], [33]. Thus, the targeting of CtBP2-S to synapses and nuclei of neuronal cells shown here might point to the function of CtBP2-S as synapto-nuclear messenger proteins.

Taken together, in this study we demonstrated that CtBP1 and CtBP2 display highly specific distribution in the adult brain, differing in their expression levels, regional and cell-specific expression patterns and in their subcellular targeting. We documented novel synaptic localization of CtBP2 in brain and cultured cells, which is restricted to the close vicinity of presynaptic active zones. Moreover, we discovered a differential targeting mode of two previously described isoforms of CtBP2 in neurons, with CtBP2-L predominantly located in cell nuclei and CtBP2-S showing dual synapto-nuclear localization. This comparative study is an attempt to pave the way for a systematic functional analysis of the differential expression of the members of CtBP family in brain and represents the first step in understanding their exclusive and overlapping functions in neuronal cells.

## Supporting Information

Figure S1 **Staining of brain slices with independently generated antibodies against CtBP1 and CtBP2.** The sagittal slices of adult mouse brain were stained with antibody from rabbit against CtBP1 (A) and from mouse against CtBP2 (B) and corresponding fluorescently coupled secondary antibodies. Please note identical staining pattern with independently raised antibodies against CtBP1 in Fig. 2A and A in this figure and with antibodies against CtBP2 in Fig. 2C and B in this figure.
(TIF)
