# Supplementary figures and images for: Differential Spatial Expression and Subcellular Localization of CtBP Family Members in Rodent Brain
Source: PLoS One. 2012 Jun 22;7(6):e39710. doi: 10.1371/journal.pone.0039710 (PMC3382178; doi:10.1371/journal.pone.0039710)

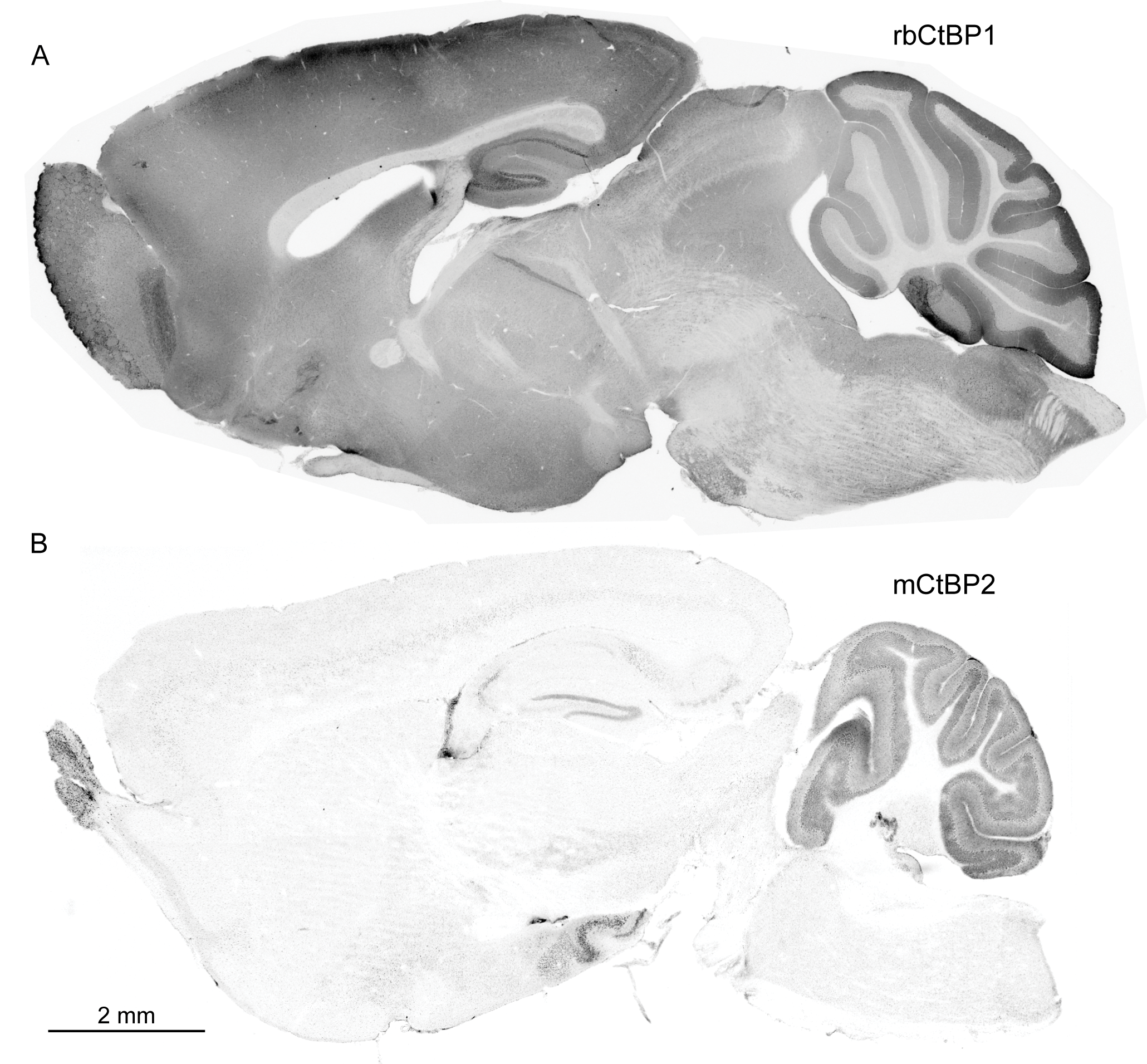

Supplement: Figure S1 — Staining of brain slices with independently generated antibodies against CtBP1 and CtBP2. The sagittal slices of adult mouse brain were stained with antibody from rabbit against CtBP1 (A) and from mouse against CtBP2 (B) and corresponding fluorescently coupled secondary antibodies. Please note identical staining pattern with independently raised antibodies against CtBP1 in Fig. 2A and A in this figure and with antibodies against CtBP2 in Fig. 2C and B in this figure. (TIF) [file pone.0039710.s001.tif]
